# Bis(μ-4-nitro­phthalato)bis­[diaqua­(1,10-phenanthroline)manganese(II)]

**DOI:** 10.1107/S1600536809024064

**Published:** 2009-07-01

**Authors:** Bi-Yi Xu, Ting Xie, Sheng-Jun Lu, Bin Xue, Wei Li

**Affiliations:** a School of Materials and Architectural Engineering, Guizhou Normal University, Guiyang 550014, People’s Republic of China; b National Engineering Research Center for Compounding and Modification of Polymeric Materials, Guiyang, Guizhou, 550014, People’s Republic of China

## Abstract

In the title compound, [Mn_2_(C_8_H_3_NO_6_)_2_(C_12_H_8_N_2_)_2_(H_2_O)_4_], the Mn^II^ atom in the centrosymmetric binuclear unit has a distorted octa­hedral geometry and is coordinated by a chelating 1,10-phenanthroline ligand, two monodentate carboxyl­ate anions from two 4-nitro­phthalates and two coordinated water mol­ecules. The two Mn^II^ ions in the mol­ecule are bridged by two 4-nitro­phthalate anions, both in a bis-monodentate mode, which finally leads to the formation of the binuclear unit. Intra­molecular O—H⋯O hydrogen bonds between the coordinated and uncoordinated O atoms of one monodentate carboxyl­ate group and the corresponding coordinated water mol­ecules result in an eight-membered and two six-membered rings. In the crystal structure, inter­molecular O—H⋯O hydrogen bonds link the dinuclear mol­ecules into supra­molecular chains propagating parallel to [100].

## Related literature

For general background to self-assembly coordination complexes with metal ions and 4-nitro­phthalic acid, see: Guo & Guo (2007 ▶); Qi *et al.* (2008 ▶).
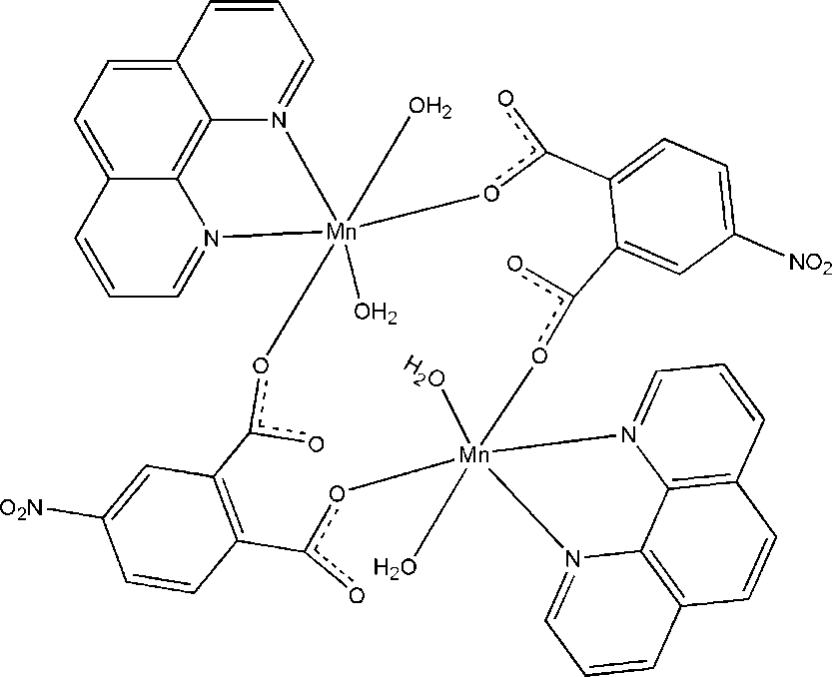


## Experimental

### 

#### Crystal data

[Mn_2_(C_8_H_3_NO_6_)_2_(C_12_H_8_N_2_)_2_(H_2_O)_4_]
*M* *_r_* = 960.58
Orthorhombic, 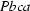

*a* = 7.1601 (9) Å
*b* = 20.039 (3) Å
*c* = 26.592 (3) Å
*V* = 3815.5 (9) Å^3^
*Z* = 4
Mo *K*α radiation
μ = 0.75 mm^−1^
*T* = 293 K
0.30 × 0.15 × 0.05 mm

#### Data collection

Bruker APEXII CCD area-detector diffractometer
Absorption correction: multi-scan (*SADABS*; Sheldrick, 1996 ▶) *T*_min_ = 0.890, *T*_max_ = 0.928
27311 measured reflections
3416 independent reflections
2608 reflections with *I* > 2σ(*I*)
*R*_int_ = 0.075

#### Refinement

*R*[*F* ^2^ > 2σ(*F* ^2^)] = 0.038
*wR*(*F* ^2^) = 0.107
*S* = 1.05
3416 reflections
305 parameters
4 restraints
H atoms treated by a mixture of independent and constrained refinement
Δρ_max_ = 0.39 e Å^−3^
Δρ_min_ = −0.34 e Å^−3^

### 

Data collection: *APEX2* (Bruker, 2007 ▶); cell refinement: *SAINT* (Bruker, 2007 ▶); data reduction: *SAINT*; program(s) used to solve structure: *SHELXS97* (Sheldrick, 2008 ▶); program(s) used to refine structure: *SHELXL97* (Sheldrick, 2008 ▶); molecular graphics: *SHELXL97* (Sheldrick, 2008 ▶); software used to prepare material for publication: *publCIF* (Westrip, 2009 ▶).

## Supplementary Material

Crystal structure: contains datablocks I, global. DOI: 10.1107/S1600536809024064/at2807sup1.cif

Structure factors: contains datablocks I. DOI: 10.1107/S1600536809024064/at2807Isup2.hkl

Additional supplementary materials:  crystallographic information; 3D view; checkCIF report

## Figures and Tables

**Table 1:** Selected bond lengths (Å)

Mn1—O3^i^	2.1212 (19)
Mn1—O1	2.1524 (18)
Mn1—O1*W*	2.1969 (19)
Mn1—O2*W*	2.2413 (19)
Mn1—N2	2.284 (2)
Mn1—N3	2.287 (2)

Symmetry code: (i) 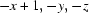.

**Table 2:** Hydrogen-bond geometry (Å, °)

*D*—H⋯*A*	*D*—H	H⋯*A*	*D*⋯*A*	*D*—H⋯*A*
O2*W*—H2*A*⋯O2	0.845 (10)	2.25 (3)	2.935 (3)	139 (3)
O2*W*—H2*B*⋯O4^ii^	0.845 (10)	2.012 (13)	2.844 (3)	167 (4)
O1*W*—H1*A*⋯O1^i^	0.845 (10)	1.896 (12)	2.732 (2)	170 (4)
O1*W*—H1*B*⋯O4^ii^	0.845 (10)	2.07 (2)	2.827 (3)	149 (3)

Symmetry codes: (i) 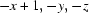; (ii) 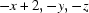.

**Fig. 1.**
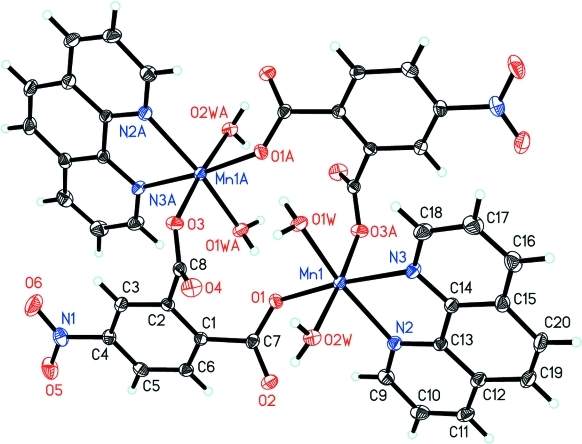
The molecular structure of the title dinuclear complex with the atom-numbering scheme. Displacement ellipsoids are drawn at the 30% probability level. [Symmetry code: (A) –x+1, –y, –z].

**Fig. 2.**
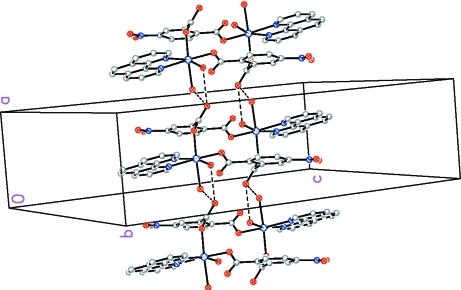
The one-dimensional supramolecular chain of the title complex. Hydrogen bonds are shown as dashed line. Hydrogen atoms are omitted for clarity.

## supplementary crystallographic information

### Comment

The self-assembly of complexes from phthalic acid ligand and transition metal ions has attracted considerable attention in recent years because these complexes have various intriguing topological structures and potential applications in material chemistry. However, only a few metal-nitrophthalate complexes have been reported to date in contrast with the abundance of metal-phthalate complexes (Guo *et al.*, 2007; Qi *et al.*, 2008). In order to enrich the metal-nitrophthalate complexes, we utilized the 4-nitrophthalic acid to assemble with manganese ions in the presence of ancillary 1,10-phenanthroline ligand and obtained the title binuclear Mn^II^ complex [Mn(1,10-phenanthroline)(C_8_H_3_NO_6_)(H_2_O)_2_]_2_.

As depicted in Fig. 1, the title complex exhibits a binuclear structure and in the dimer each Mn^II^ ion has a distorted octahedral geometry and was coordinated by a chelating 1,10-phenanthroline, two monodentate carboxylates from two 4-nitrophthalates and two coordinated water molecules. And it is noteworthy that the two Mn^II^ ions in the complex are bridged by two 4-nitrophthalates both in a bis-monodentate mode to lead to the formation of a dinuclear unit because of the presence of an inversion center in the crystal structure. Intramolecular O—H···O hydrogen bonds between the coordinated and uncoordinated oxygen atoms of one monodentate carboxylate in a 4-nitrophthalate and corresponding coordinated water molecules result in an eight-membered and two six-membered rings (Table 2). Furthermore, the intermolecular O—H···O hydrogen bonds between two water molecules and another monodentate carboxylate in the same 4-nitrophthalate link the dinuclear molecules into a one-dimensional supramolecular chain, as shown in Fig. 2.

### Experimental

Mn(CH_3_COO)_2_.4H_2_O (0.50 mmol, 0.122 g), 4-nitrophthalic acid (0.50 mmol, 0.103 g), 1,10-phenanthroline (0.50 mmol, 0.099 g) and NaOH (1.0 mmol, 0.040 g) were well mixed in 8 ml distilled water, and the solution was stirred for 15 min and then transferred into a 23 ml Teflon-lined bomb at 398 K for 3 days and slowly cooled to room temperature. Light yellow sheet crystals which were suitable for X-ray analysis were obtained.

### Refinement

H atoms of water molecules were located in difference Fourier maps and refined isotropically with restraints of O1W—H1A = 0.845 (10), O1W—H1B = 0.846 (10), O2W—H2A = 0.845 (10), O2W—H2B = 0.846 (10) Å and H1A—O1W—H1B = 107 (3) and H2A—O2W—H2B = 112 (4)°. The remaining H atoms of aromatic rings were positioned geometrically with C—H = 0.95 Å and constrained to ride on their parent atoms with *U*_iso_(H) = 1.2U_eq_(C).

### Crystal data

**Table:**

[Mn_2_(C_8_H_3_NO_6_)_2_(C_12_H_8_N_2_)_2_(H_2_O)_4_]	*F*(000) = 1960
*M**_r_* = 960.58	*D*_x_ = 1.672 Mg m^−^^3^
Orthorhombic, *P**b**c**a*	Mo *K*α radiation, λ = 0.71073 Å
Hall symbol: -P 2ac 2ab	Cell parameters from 4478 reflections
*a* = 7.1601 (9) Å	θ = 2.5–23.3°
*b* = 20.039 (3) Å	µ = 0.75 mm^−^^1^
*c* = 26.592 (3) Å	*T* = 293 K
*V* = 3815.5 (9) Å^3^	Sheet, yellow
*Z* = 4	0.30 × 0.15 × 0.05 mm

### Data collection

**Table:**

Bruker APEXII CCD area-detector diffractometer	3416 independent reflections
Radiation source: fine-focus sealed tube	2608 reflections with *I* > 2σ(*I*)
graphite	*R*_int_ = 0.075
φ and ω scans	θ_max_ = 25.2°, θ_min_ = 1.5°
Absorption correction: multi-scan (*SADABS*; Sheldrick, 1996)	*h* = −8→8
*T*_min_ = 0.890, *T*_max_ = 0.928	*k* = −23→22
27311 measured reflections	*l* = −31→31

### Refinement

**Table:**

Refinement on *F*^2^	Primary atom site location: structure-invariant direct methods
Least-squares matrix: full	Secondary atom site location: difference Fourier map
*R*[*F*^2^ > 2σ(*F*^2^)] = 0.038	Hydrogen site location: inferred from neighbouring sites
*wR*(*F*^2^) = 0.107	H atoms treated by a mixture of independent and constrained refinement
*S* = 1.05	*w* = 1/[σ^2^(*F*_o_^2^) + (0.06*P*)^2^ + 0.8459*P*] where *P* = (*F*_o_^2^ + 2*F*_c_^2^)/3
3416 reflections	(Δ/σ)_max_ = 0.001
305 parameters	Δρ_max_ = 0.39 e Å^−^^3^
4 restraints	Δρ_min_ = −0.34 e Å^−^^3^

### Special details

**Table:**

Geometry. All e.s.d.'s (except the e.s.d. in the dihedral angle between two l.s. planes) are estimated using the full covariance matrix. The cell e.s.d.'s are taken into account individually in the estimation of e.s.d.'s in distances, angles and torsion angles; correlations between e.s.d.'s in cell parameters are only used when they are defined by crystal symmetry. An approximate (isotropic) treatment of cell e.s.d.'s is used for estimating e.s.d.'s involving l.s. planes.
Refinement. Refinement of *F*^2^ against ALL reflections. The weighted *R*-factor *wR* and goodness of fit *S* are based on *F*^2^, conventional *R*-factors *R* are based on *F*, with *F* set to zero for negative *F*^2^. The threshold expression of *F*^2^ > σ(*F*^2^) is used only for calculating *R*-factors(gt) *etc*. and is not relevant to the choice of reflections for refinement. *R*-factors based on *F*^2^ are statistically about twice as large as those based on *F*, and *R*- factors based on ALL data will be even larger.

### Fractional atomic coordinates and isotropic or equivalent isotropic displacement parameters (Å^2^)

**Table:**

	*x*	*y*	*z*	*U*_iso_*/*U*_eq_	
Mn1	0.60706 (5)	−0.01188 (2)	0.092001 (13)	0.02468 (15)	
O1	0.5576 (2)	0.07756 (9)	0.04921 (6)	0.0285 (4)	
O3	0.6721 (3)	0.04306 (9)	−0.07937 (7)	0.0316 (4)	
O4	0.9042 (3)	0.06002 (10)	−0.02534 (7)	0.0373 (5)	
O1W	0.7016 (3)	−0.06823 (10)	0.02586 (7)	0.0319 (5)	
O2	0.7356 (3)	0.15076 (10)	0.09074 (7)	0.0426 (5)	
O2W	0.9075 (3)	0.01824 (10)	0.09875 (7)	0.0313 (5)	
O5	0.6617 (3)	0.36361 (11)	−0.11306 (9)	0.0513 (6)	
N3	0.6745 (3)	−0.10062 (11)	0.14296 (7)	0.0280 (5)	
N2	0.5723 (3)	0.02382 (11)	0.17303 (8)	0.0273 (5)	
N1	0.6741 (3)	0.30363 (12)	−0.11986 (10)	0.0405 (6)	
C13	0.6103 (3)	−0.02215 (12)	0.20908 (9)	0.0228 (6)	
C2	0.6986 (3)	0.14993 (12)	−0.04068 (9)	0.0230 (6)	
C7	0.6488 (4)	0.13194 (13)	0.05290 (9)	0.0260 (6)	
C12	0.5995 (3)	−0.00806 (14)	0.26127 (10)	0.0298 (6)	
C14	0.6635 (3)	−0.08850 (13)	0.19318 (9)	0.0258 (6)	
C1	0.6516 (3)	0.17594 (13)	0.00669 (9)	0.0232 (6)	
C5	0.6125 (4)	0.28622 (14)	−0.02963 (10)	0.0298 (6)	
H5	0.5839	0.3313	−0.0263	0.036*	
C3	0.7018 (4)	0.19241 (14)	−0.08173 (9)	0.0285 (6)	
H3	0.7312	0.1758	−0.1134	0.034*	
C6	0.6077 (3)	0.24341 (13)	0.01140 (10)	0.0267 (6)	
H6	0.5744	0.2602	0.0428	0.032*	
C11	0.5484 (4)	0.05711 (15)	0.27502 (10)	0.0347 (7)	
H11	0.5400	0.0688	0.3088	0.042*	
C15	0.7010 (4)	−0.13834 (14)	0.22946 (10)	0.0306 (6)	
C8	0.7626 (4)	0.07836 (12)	−0.04821 (9)	0.0244 (6)	
C10	0.5110 (4)	0.10331 (15)	0.23861 (11)	0.0373 (7)	
H10	0.4772	0.1466	0.2474	0.045*	
C9	0.5241 (4)	0.08472 (13)	0.18791 (10)	0.0327 (6)	
H9	0.4979	0.1166	0.1635	0.039*	
C4	0.6617 (4)	0.25922 (13)	−0.07580 (10)	0.0285 (6)	
C19	0.6417 (4)	−0.05922 (16)	0.29694 (10)	0.0370 (7)	
H19	0.6359	−0.0497	0.3311	0.044*	
C17	0.7581 (4)	−0.21389 (15)	0.16140 (11)	0.0428 (7)	
H17	0.7888	−0.2561	0.1494	0.051*	
C20	0.6903 (4)	−0.12145 (16)	0.28170 (10)	0.0376 (7)	
H20	0.7171	−0.1538	0.3057	0.045*	
C18	0.7211 (4)	−0.16194 (14)	0.12807 (11)	0.0362 (7)	
H18	0.7293	−0.1704	0.0938	0.043*	
C16	0.7488 (4)	−0.20209 (14)	0.21190 (11)	0.0407 (7)	
H16	0.7740	−0.2362	0.2346	0.049*	
O6	0.6997 (5)	0.27861 (13)	−0.16128 (8)	0.0716 (8)	
H2A	0.918 (5)	0.0602 (6)	0.0986 (13)	0.061 (12)*	
H2B	0.976 (5)	−0.0003 (17)	0.0769 (11)	0.067 (12)*	
H1A	0.632 (4)	−0.0697 (19)	0.0003 (9)	0.073 (13)*	
H1B	0.809 (2)	−0.0579 (18)	0.0155 (13)	0.066 (12)*	

### Atomic displacement parameters (Å^2^)

**Table:**

	*U*^11^	*U*^22^	*U*^33^	*U*^12^	*U*^13^	*U*^23^
Mn1	0.0297 (2)	0.0251 (3)	0.0193 (2)	0.00034 (16)	−0.00131 (15)	0.00056 (15)
O1	0.0308 (10)	0.0279 (11)	0.0269 (10)	−0.0050 (8)	−0.0032 (7)	0.0050 (8)
O3	0.0317 (10)	0.0265 (11)	0.0365 (11)	−0.0016 (8)	0.0007 (8)	−0.0079 (8)
O4	0.0309 (11)	0.0372 (12)	0.0438 (12)	0.0112 (9)	−0.0071 (9)	−0.0026 (9)
O1W	0.0303 (12)	0.0402 (13)	0.0252 (11)	−0.0035 (9)	−0.0001 (9)	−0.0074 (9)
O2	0.0658 (15)	0.0358 (12)	0.0261 (11)	−0.0102 (11)	−0.0095 (10)	−0.0010 (8)
O2W	0.0310 (11)	0.0314 (13)	0.0315 (11)	0.0004 (9)	−0.0026 (8)	−0.0031 (9)
O5	0.0666 (15)	0.0287 (13)	0.0587 (14)	0.0087 (10)	0.0136 (12)	0.0166 (10)
N3	0.0328 (12)	0.0301 (13)	0.0212 (11)	0.0041 (10)	−0.0001 (9)	−0.0022 (9)
N2	0.0275 (12)	0.0294 (13)	0.0249 (12)	−0.0005 (9)	0.0007 (9)	−0.0004 (9)
N1	0.0475 (15)	0.0311 (16)	0.0430 (16)	0.0012 (11)	0.0003 (12)	0.0127 (12)
C13	0.0201 (13)	0.0266 (15)	0.0218 (13)	−0.0017 (10)	−0.0003 (10)	−0.0020 (10)
C2	0.0237 (13)	0.0208 (14)	0.0244 (13)	−0.0009 (10)	−0.0021 (10)	0.0007 (10)
C7	0.0301 (14)	0.0263 (15)	0.0217 (14)	0.0033 (12)	0.0033 (11)	−0.0010 (11)
C12	0.0223 (13)	0.0428 (18)	0.0243 (15)	−0.0040 (12)	−0.0010 (10)	−0.0012 (12)
C14	0.0230 (13)	0.0309 (15)	0.0234 (14)	−0.0026 (11)	0.0001 (10)	0.0010 (11)
C1	0.0218 (13)	0.0250 (15)	0.0228 (13)	−0.0013 (10)	−0.0005 (10)	0.0002 (10)
C5	0.0283 (14)	0.0224 (15)	0.0387 (16)	0.0000 (11)	0.0004 (11)	−0.0009 (12)
C3	0.0345 (15)	0.0292 (16)	0.0217 (13)	−0.0008 (12)	0.0010 (11)	0.0013 (11)
C6	0.0264 (13)	0.0265 (15)	0.0272 (14)	−0.0007 (11)	0.0013 (10)	−0.0040 (11)
C11	0.0338 (15)	0.0428 (18)	0.0276 (15)	−0.0040 (13)	0.0030 (12)	−0.0110 (13)
C15	0.0271 (14)	0.0355 (17)	0.0293 (15)	−0.0032 (12)	−0.0035 (11)	0.0072 (12)
C8	0.0240 (13)	0.0251 (14)	0.0241 (13)	−0.0002 (11)	0.0057 (11)	−0.0008 (11)
C10	0.0364 (16)	0.0346 (17)	0.0409 (17)	−0.0008 (13)	0.0060 (13)	−0.0146 (13)
C9	0.0318 (15)	0.0289 (16)	0.0373 (16)	0.0021 (12)	0.0014 (12)	−0.0007 (12)
C4	0.0324 (14)	0.0262 (16)	0.0270 (14)	0.0013 (11)	−0.0024 (11)	0.0056 (11)
C19	0.0377 (17)	0.053 (2)	0.0202 (14)	−0.0070 (14)	0.0001 (11)	0.0009 (13)
C17	0.0522 (19)	0.0273 (16)	0.0489 (19)	0.0080 (14)	−0.0047 (14)	−0.0019 (14)
C20	0.0400 (17)	0.048 (2)	0.0249 (15)	−0.0035 (14)	−0.0047 (12)	0.0123 (13)
C18	0.0451 (17)	0.0308 (17)	0.0325 (16)	0.0062 (13)	−0.0013 (12)	−0.0073 (12)
C16	0.0436 (17)	0.0333 (18)	0.0452 (18)	0.0032 (14)	−0.0067 (14)	0.0111 (14)
O6	0.137 (3)	0.0484 (15)	0.0296 (13)	−0.0022 (16)	0.0067 (13)	0.0073 (11)

### Geometric parameters (Å, °)

**Table:**

Mn1—O3^i^	2.1212 (19)	C7—C1	1.513 (3)
Mn1—O1	2.1524 (18)	C12—C11	1.405 (4)
Mn1—O1W	2.1969 (19)	C12—C19	1.429 (4)
Mn1—O2W	2.2413 (19)	C14—C15	1.414 (4)
Mn1—N2	2.284 (2)	C1—C6	1.394 (4)
Mn1—N3	2.287 (2)	C5—C4	1.387 (4)
O1—C7	1.274 (3)	C5—C6	1.388 (4)
O3—C8	1.267 (3)	C5—H5	0.9300
O3—Mn1^i^	2.1212 (19)	C3—C4	1.378 (4)
O4—C8	1.238 (3)	C3—H3	0.9300
O1W—H1A	0.845 (10)	C6—H6	0.9300
O1W—H1B	0.845 (10)	C11—C10	1.366 (4)
O2—C7	1.241 (3)	C11—H11	0.9300
O2W—H2A	0.845 (10)	C15—C16	1.402 (4)
O2W—H2B	0.845 (10)	C15—C20	1.432 (4)
O5—N1	1.219 (3)	C10—C9	1.402 (4)
N3—C18	1.333 (3)	C10—H10	0.9300
N3—C14	1.360 (3)	C9—H9	0.9300
N2—C9	1.329 (3)	C19—C20	1.356 (4)
N2—C13	1.357 (3)	C19—H19	0.9300
N1—O6	1.224 (3)	C17—C16	1.365 (4)
N1—C4	1.474 (3)	C17—C18	1.393 (4)
C13—C12	1.418 (4)	C17—H17	0.9300
C13—C14	1.446 (4)	C20—H20	0.9300
C2—C3	1.385 (4)	C18—H18	0.9300
C2—C1	1.404 (3)	C16—H16	0.9300
C2—C8	1.519 (3)		
			
O3^i^—Mn1—O1	90.38 (7)	C15—C14—C13	120.0 (2)
O3^i^—Mn1—O1W	90.70 (7)	C6—C1—C2	119.7 (2)
O1—Mn1—O1W	93.18 (7)	C6—C1—C7	119.3 (2)
O3^i^—Mn1—O2W	175.18 (7)	C2—C1—C7	121.0 (2)
O1—Mn1—O2W	88.61 (7)	C4—C5—C6	117.4 (3)
O1W—Mn1—O2W	84.66 (7)	C4—C5—H5	121.3
O3^i^—Mn1—N2	97.98 (7)	C6—C5—H5	121.3
O1—Mn1—N2	102.71 (7)	C4—C3—C2	120.2 (2)
O1W—Mn1—N2	161.78 (8)	C4—C3—H3	119.9
O2W—Mn1—N2	86.83 (7)	C2—C3—H3	119.9
O3^i^—Mn1—N3	93.66 (8)	C5—C6—C1	121.5 (2)
O1—Mn1—N3	174.46 (7)	C5—C6—H6	119.2
O1W—Mn1—N3	90.56 (7)	C1—C6—H6	119.2
O2W—Mn1—N3	87.67 (8)	C10—C11—C12	119.8 (3)
N2—Mn1—N3	73.00 (7)	C10—C11—H11	120.1
C7—O1—Mn1	125.97 (16)	C12—C11—H11	120.1
C8—O3—Mn1^i^	138.54 (16)	C16—C15—C14	117.5 (2)
Mn1—O1W—H1A	119 (3)	C16—C15—C20	123.5 (3)
Mn1—O1W—H1B	115 (3)	C14—C15—C20	119.0 (3)
H1A—O1W—H1B	107 (3)	O4—C8—O3	125.0 (2)
Mn1—O2W—H2A	111 (3)	O4—C8—C2	117.5 (2)
Mn1—O2W—H2B	113 (3)	O3—C8—C2	117.3 (2)
H2A—O2W—H2B	112 (4)	C11—C10—C9	119.2 (3)
C18—N3—C14	118.1 (2)	C11—C10—H10	120.4
C18—N3—Mn1	126.39 (17)	C9—C10—H10	120.4
C14—N3—Mn1	115.52 (17)	N2—C9—C10	123.2 (3)
C9—N2—C13	117.7 (2)	N2—C9—H9	118.4
C9—N2—Mn1	126.66 (18)	C10—C9—H9	118.4
C13—N2—Mn1	115.60 (16)	C3—C4—C5	122.2 (2)
O5—N1—O6	123.3 (2)	C3—C4—N1	118.9 (2)
O5—N1—C4	118.2 (2)	C5—C4—N1	118.9 (2)
O6—N1—C4	118.5 (2)	C20—C19—C12	121.0 (3)
N2—C13—C12	123.0 (2)	C20—C19—H19	119.5
N2—C13—C14	118.0 (2)	C12—C19—H19	119.5
C12—C13—C14	118.9 (2)	C16—C17—C18	119.2 (3)
C3—C2—C1	118.9 (2)	C16—C17—H17	120.4
C3—C2—C8	118.1 (2)	C18—C17—H17	120.4
C1—C2—C8	122.8 (2)	C19—C20—C15	121.4 (3)
O2—C7—O1	125.4 (2)	C19—C20—H20	119.3
O2—C7—C1	118.4 (2)	C15—C20—H20	119.3
O1—C7—C1	116.3 (2)	N3—C18—C17	123.2 (3)
C11—C12—C13	117.0 (2)	N3—C18—H18	118.4
C11—C12—C19	123.3 (3)	C17—C18—H18	118.4
C13—C12—C19	119.7 (3)	C17—C16—C15	119.8 (3)
N3—C14—C15	122.2 (2)	C17—C16—H16	120.1
N3—C14—C13	117.8 (2)	C15—C16—H16	120.1
			
O3^i^—Mn1—O1—C7	156.7 (2)	C8—C2—C1—C7	−4.5 (4)
O1W—Mn1—O1—C7	−112.6 (2)	O2—C7—C1—C6	−50.6 (3)
O2W—Mn1—O1—C7	−28.0 (2)	O1—C7—C1—C6	130.2 (2)
N2—Mn1—O1—C7	58.4 (2)	O2—C7—C1—C2	129.1 (3)
N3—Mn1—O1—C7	19.8 (9)	O1—C7—C1—C2	−50.2 (3)
O3^i^—Mn1—N3—C18	81.4 (2)	C1—C2—C3—C4	0.8 (4)
O1—Mn1—N3—C18	−141.8 (7)	C8—C2—C3—C4	−174.0 (2)
O1W—Mn1—N3—C18	−9.4 (2)	C4—C5—C6—C1	0.4 (4)
O2W—Mn1—N3—C18	−94.0 (2)	C2—C1—C6—C5	−1.2 (4)
N2—Mn1—N3—C18	178.6 (2)	C7—C1—C6—C5	178.4 (2)
O3^i^—Mn1—N3—C14	−98.02 (18)	C13—C12—C11—C10	0.3 (4)
O1—Mn1—N3—C14	38.8 (9)	C19—C12—C11—C10	179.7 (3)
O1W—Mn1—N3—C14	171.24 (18)	N3—C14—C15—C16	−0.8 (4)
O2W—Mn1—N3—C14	86.61 (18)	C13—C14—C15—C16	178.9 (2)
N2—Mn1—N3—C14	−0.79 (17)	N3—C14—C15—C20	178.7 (2)
O3^i^—Mn1—N2—C9	−89.3 (2)	C13—C14—C15—C20	−1.6 (4)
O1—Mn1—N2—C9	2.9 (2)	Mn1^i^—O3—C8—O4	127.8 (2)
O1W—Mn1—N2—C9	153.0 (2)	Mn1^i^—O3—C8—C2	−55.9 (3)
O2W—Mn1—N2—C9	90.8 (2)	C3—C2—C8—O4	115.6 (3)
N3—Mn1—N2—C9	179.3 (2)	C1—C2—C8—O4	−59.0 (3)
O3^i^—Mn1—N2—C13	92.61 (17)	C3—C2—C8—O3	−61.0 (3)
O1—Mn1—N2—C13	−175.18 (16)	C1—C2—C8—O3	124.5 (3)
O1W—Mn1—N2—C13	−25.1 (3)	C12—C11—C10—C9	0.1 (4)
O2W—Mn1—N2—C13	−87.32 (17)	C13—N2—C9—C10	0.0 (4)
N3—Mn1—N2—C13	1.20 (16)	Mn1—N2—C9—C10	−178.07 (19)
C9—N2—C13—C12	0.4 (3)	C11—C10—C9—N2	−0.2 (4)
Mn1—N2—C13—C12	178.70 (17)	C2—C3—C4—C5	−1.7 (4)
C9—N2—C13—C14	−179.8 (2)	C2—C3—C4—N1	177.5 (2)
Mn1—N2—C13—C14	−1.5 (3)	C6—C5—C4—C3	1.0 (4)
Mn1—O1—C7—O2	−25.1 (4)	C6—C5—C4—N1	−178.1 (2)
Mn1—O1—C7—C1	154.09 (16)	O5—N1—C4—C3	−170.9 (3)
N2—C13—C12—C11	−0.6 (4)	O6—N1—C4—C3	7.8 (4)
C14—C13—C12—C11	179.6 (2)	O5—N1—C4—C5	8.3 (4)
N2—C13—C12—C19	−180.0 (2)	O6—N1—C4—C5	−173.0 (3)
C14—C13—C12—C19	0.2 (3)	C11—C12—C19—C20	179.9 (3)
C18—N3—C14—C15	0.5 (4)	C13—C12—C19—C20	−0.7 (4)
Mn1—N3—C14—C15	179.98 (19)	C12—C19—C20—C15	0.0 (4)
C18—N3—C14—C13	−179.1 (2)	C16—C15—C20—C19	−179.4 (3)
Mn1—N3—C14—C13	0.3 (3)	C14—C15—C20—C19	1.2 (4)
N2—C13—C14—N3	0.8 (3)	C14—N3—C18—C17	0.2 (4)
C12—C13—C14—N3	−179.4 (2)	Mn1—N3—C18—C17	−179.2 (2)
N2—C13—C14—C15	−178.9 (2)	C16—C17—C18—N3	−0.7 (5)
C12—C13—C14—C15	0.9 (3)	C18—C17—C16—C15	0.5 (4)
C3—C2—C1—C6	0.6 (3)	C14—C15—C16—C17	0.2 (4)
C8—C2—C1—C6	175.1 (2)	C20—C15—C16—C17	−179.2 (3)
C3—C2—C1—C7	−179.0 (2)		

Symmetry codes: (i) −*x*+1, −*y*, −*z*.

### Hydrogen-bond geometry (Å, °)

**Table:**

*D*—H···*A*	*D*—H	H···*A*	*D*···*A*	*D*—H···*A*
O2W—H2A···O2	0.85 (1)	2.25 (3)	2.935 (3)	139 (3)
O2W—H2B···O4^ii^	0.85 (1)	2.01 (1)	2.844 (3)	167 (4)
O1W—H1A···O1^i^	0.85 (1)	1.90 (1)	2.732 (2)	170 (4)
O1W—H1B···O4^ii^	0.85 (1)	2.07 (2)	2.827 (3)	149 (3)

Symmetry codes: (ii) −*x*+2, −*y*, −*z*; (i) −*x*+1, −*y*, −*z*.
